# Measurement Properties of the NeuroFlexor Device for Quantifying Neural and Non-neural Components of Wrist Hyper-Resistance in Chronic Stroke

**DOI:** 10.3389/fneur.2019.00730

**Published:** 2019-07-03

**Authors:** Aukje Andringa, Erwin van Wegen, Ingrid van de Port, Gert Kwakkel, Carel Meskers

**Affiliations:** ^1^Department of Rehabilitation Medicine, Amsterdam Movement Sciences, Amsterdam Neuroscience, Amsterdam UMC, Vrije Universiteit Amsterdam, Amsterdam, Netherlands; ^2^Revant Rehabilitation Centre Breda, Breda, Netherlands; ^3^Department of Physical Therapy and Human Movement Sciences, Northwestern University, Chicago, IL, United States; ^4^Department of Neurorehabilitation, Amsterdam Rehabilitation Research Centre, Reade, Amsterdam, Netherlands

**Keywords:** reliability, validity, assessment, biomechanics, stroke, spasticity

## Abstract

**Introduction:** Differentiating between the components of wrist hyper-resistance post stroke, i.e., pathological neuromuscular activation (“spasticity”) and non-neural biomechanical changes, is important for treatment decisions. This study aimed to assess the reliability and construct validity of an innovative measurement device that quantifies these neural and non-neural components by biomechanical modeling.

**Methods:** Forty-six patients with chronic stroke and 30 healthy age-matched subjects were assessed with the NeuroFlexor, a motor-driven device that imposes isokinetic wrist extensions at two controlled velocities (5 and 236°/s). Test-retest reliability was evaluated using intraclass correlation coefficients (ICC) and smallest detectable changes (SDC), and construct validity by testing the difference between patients and healthy subjects and between subgroups of patients stratified by modified Ashworth scale (MAS), and the association with clinical scales.

**Results:** Test-retest reliability was excellent for the neural (NC) and non-neural elastic (EC) components (ICC 0.93 and 0.95, respectively), and good for the viscous component (VC) (ICC 0.84), with SDCs of 10.3, 3.1, and 0.5 N, respectively. NC and EC were significantly higher in patients compared to healthy subjects (*p* < 0.001). Components gradually increased with MAS category. NC and EC were positively associated with the MAS (*r*_s_ 0.60 and 0.52, respectively; *p* < 0.01), and NC with the Tardieu scale (*r*_s_ 0.36, *p* < 0.05). NC and EC were negatively associated with the Fugl-Meyer Assessment of the upper extremity and action research arm test (*r*_s_ ≤ −0.38, *p* < 0.05).

**Conclusions:** The NeuroFlexor reliably quantifies neural and non-neural components of wrist hyper-resistance in chronic stroke, but is less suitable for clinical evaluation at individual level due to high SDC values. Although construct validity has been demonstrated, further investigation at component level is needed.

## Introduction

Hyper-resistance in the wrist joint after stroke is a result of pathological neuromuscular activation (“spasticity”) and biomechanical changes in muscles and soft tissues overlying the joint (1–3). Distribution and level of these neural and non-neural components may diverge between individual patients, and may change during the time course post stroke (4, 5). Distinguishing between components will impact on the choice of tailored interventions for the prevention and treatment of joint hyper-resistance.

The modified Ashworth scale (MAS) is routinely used as a clinical measurement scale for spasticity, as it is easily applicable, time-efficient and cost-free. However, this ordinal rating scale has poor measurement properties regarding reliability (6–8) and validity (6, 8–10), and is unable to discriminate between spasticity and other factors influencing joint hyper-resistance. There is a need for an objective, quantitative measurement tool, with a standardized assessment protocol, feasible for clinical practice, which is reliable and valid. In recent years, various instrumented measurement setups using different modeling techniques were developed (11–14). However, these are generally time-consuming and require extensive training. The NeuroFlexor (Aggero MedTech AB, Älta, Sweden) is a recently developed, portable, easily applicable and commercially available alternative. The underlying biomechanical model for the quantification of the neural component (“spasticity”) was previously validated (4). Good inter- and intratester reliability for both neural and non-neural components has been demonstrated for patients with chronic stroke (15). However, all studies of the measurement properties of the NeuroFlexor so far have been published by authors who potentially have commercial interest in the device. Furthermore, information regarding the validity of the different components compared to commonly used clinical scales is lacking.

Therefore, the aim of this study is to perform an independent investigation of the reliability and construct validity of the NeuroFlexor for the quantification of neural and non-neural components of wrist hyper-resistance in patients with chronic stroke.

## Materials and Methods

### Participants

We recruited patients with chronic stroke from Revant rehabilitation center Breda, Klimmendaal Rehabilitation center Apeldoorn, Bravis hospital Bergen op Zoom and Roosendaal, and from physiotherapists of the stroke network Amsterdam and FysioNet Breda. The inclusion criteria for this study were: (1) an ischemic or hemorrhagic stroke at least 6 months prior to inclusion; (2) an initial upper limb deficit as defined by the National Institutes of Health Stroke Scale (NIHSS) item 5 a/b score > 0 (i.e., not able to hold the affected arm at a 90° angle for at least 10 s); (3) age ≥ 18 years; and (4) the ability to follow test instructions (mini mental state examination (MMSE) > 19). Exclusion criteria were: (1) limitations of arm-hand function of the affected side other than due to stroke; (2) limitation of the wrist passive range of motion (pROM) with extended fingers that limits the extension to <40°; and (3) botulinum toxin injections in the affected arm in the previous 3 months. A group of right-handed healthy age-matched adults without wrist function restrictions volunteered as a reference group. Ethical approval was obtained from the Medical Ethics Committee of the VU University medical center, Amsterdam, The Netherlands. In accordance with the Declaration of Helsinki (2013), all participants gave written informed consent.

### Outcome Measures

#### NeuroFlexor

**Figure 1:**
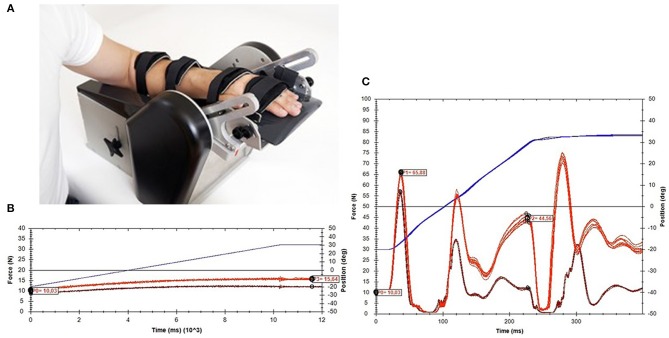
NeuroFlexor method. **(A)** Measurement set-up. **(B)** An example of data obtained during slow movements (5°/s). **(C)** An example of data obtained during fast movements (236°/s).

The NeuroFlexor (Aggero MedTech AB, Älta, Sweden) is a motor-driven device that imposes isokinetic displacements on the wrist with extended fingers in the direction of extension, at two controlled velocities (5 and 236°/s) as pictured in Figure 1. Resistance during the passive movement is measured in Newton (N) using a force sensor, which is placed underneath the moveable hand platform. The resulting resistance trace during the displacement is subsequently analyzed by a biomechanical model, which results in quantification of the different components of joint resistance, i.e., the neural component (NC), elastic component (EC), and viscous component (VC) (4). The NC represents the velocity-dependent force due to muscle contractions induced by the stretch reflexes. The non-neural component consists of an elastic and a viscous component. The EC is the length-dependent force, assessed 1 s after the end of the slow movement. The VC is velocity-dependent and is most prominent during initial acceleration. During wrist extension movement with extended fingers, both the wrist flexor muscles, as well as the finger flexor muscles were lengthened. The neural and non-neural values of the NeuroFlexor, therefore, represent a combination of wrist and finger flexor muscle groups.

During the measurement, the participant was seated comfortably parallel to the device with the shoulder in 45° abduction and 0° flexion, the elbow in 90° flexion, the forearm in pronation, and the fingers extended. The arm rested in a support and was fastened to the device using two straps for the forearm and two straps for the hand and fingers, to minimize displacements during measurement. The wrist joint was visually aligned to the rotation axis, and the hand was placed on the hand platform in a standardized way according to anatomical landmarks. The participant was instructed to relax the arm during the movements of the device. The device imposed wrist joint displacements from 30° wrist flexion to 20° wrist extension. A test session consisted of five slow movements followed by 10 fast movements with a pause of at least 1 s in between the movements. In order to avoid bias from startle reflexes and mechanical hysteresis, the first slow and first fast movements were excluded from the analysis. The NeuroFlexor Scientific v0.06 software program automatically calculated the different components of joint resistance using the biomechanical model described by Lindberg et al. (4) (Supplementary File 1).

#### Clinical Assessment

Total resistance to passive movement in the wrist was measured manually using the ordinal MAS (16), which ranges from 0, indicating no increased tone, to 4, indicating that the joint is rigid. The Tardieu scale (TS) (17), which has been suggested to be more appropriate for the measurement of velocity-dependent spasticity, was used to assess the pROM at slow velocity (R2) without the effect of stretch reflex hyperactivity, the joint angle of muscle reaction at fast velocity stretch when the overactive stretch reflex produces a first catch (R1), and the quality of the muscle response at fast speed (Q). The quality of the muscle response at fast speed is described on an ordinal five-point scale, where 0 means no resistance to passive motion, and 4 means a clonus that does not cease within 10 s. The MAS and TS were performed for the wrist and finger flexor muscles separately. The wrist extension movement with extended fingers was used as a representation of the resistance mostly caused by the finger flexors muscles, while the wrist extension movement with flexed fingers represented the resistance mostly caused by the wrist flexor muscles. pROM of the wrist was determined using a goniometer. The mean of three pROM assessments was used for the validation analysis. The Fugl-Meyer motor assessment of the upper extremity (FM-UE) (18) was used to assess motor performance of the affected arm and hand, and the action research arm test (ARAT) (19) was used to assess arm and hand capacity. Both the FM-UE and ARAT have been shown to be reliable and valid tests (20–22).

### Procedure

We used a test-retest design within a cross-sectional cohort with a single experimental session. First, demographic data, medical history, type of stroke, time post stroke, neurological status (NIHSS), cognitive function (MMSE), affected body side, and hand dominance were recorded. All measurements were done by a trained researcher, and were performed on the patients' impaired arm and on the dominant right arm of the healthy subjects. To determine test-retest reliability, NeuroFlexor measurements were performed twice within the single experimental session. To achieve stable levels of hyper-resistance, the environment was quiet and no great physical effort was required from the patient in between the tests. The two NeuroFlexor measurements and the clinical assessments were performed in a random order to avoid systematic influence of the clinical assessments on the test-retest values, with at least 15 min between the two NeuroFlexor measurements, during which interval the participants' arm was removed from the device and then replaced anew.

### Statistical Analysis

All analyses were performed using IBM SPSS Statistics for Windows, version 22.0 (IBM Corp., Armonk, NY, USA). Descriptive statistics were used for demographic and clinical characteristics. We used the Consensus-based Standards for the selection of health Measurement Instruments (COSMIN) guidelines regarding definitions of reliability and validity (23).

Test-retest reliability of the NeuroFlexor was defined as the extent to which scores for patients with unchanged impairments were the same in two repeated measurements (23, 24). First, scatterplots were used to obtain a visual overview of the distribution of test-retest data, and to check for potential outliers. Test-retest reliability was evaluated using intraclass correlation coefficients (ICC), which were calculated with a single-measures, two-way random-effects model for absolute agreement with 95% confident intervals. Following Portney and Watkins' recommendations, ICC values < 0.50 were considered to indicate poor, 0.50–0.75 moderate, 0.75–0.90 good, and values > 0.90 excellent reliability (25).

To evaluate measurement error we obtained Bland-Altman plots (mean of measurements 1 and 2 [x-axis] compared with the difference between the two measurements [y-axis]) with limits of agreement, standard errors of measurement (SEM), and smallest detectable changes (SDC). Limits of agreement were calculated based on the standard deviation of the mean difference between measurements 1 and 2 (*d* ± 1.96 ^*^ SD Δ). SEM was calculated from the square root of the within-subject variance (i.e., the sum of the between-measurements variance and the residual variance), and SDC was calculated using the formula: SDC = 1.96 ^*^ √2 ^*^ SEM (24). SDC was defined as the smallest change in score that can be detected by the device and can be interpreted as a real change, which is important for use in clinical practice.

Due to the lack of an appropriate golden standard, validity was assessed in terms of construct validity. Prior hypotheses were formulated stating the expected relation between the NeuroFlexor and clinical scales. We expected (1) significantly higher neural and non-neural components in patients compared to healthy subjects, (2) positive associations between the total resistance to passive movement and the MAS scores of the wrist and fingers flexor muscles and (3) between the neural component and the scores on the Tardieu Scale, and (4) negative associations between the non-neural elastic component and wrist pROM and (5) between both the neural and non-neural components and the motor performance of the arm.

Statistical analysis of the difference in neural and non-neural components between patients and healthy subjects used the Mann-Whitney U test. Stratification by MAS score of patients was based on the highest MAS value for the wrist or the finger flexor muscles. Differences between patients, stratified by MAS, and healthy subjects were assessed by Kruskal–Wallis analysis, with Mann-Whitney U *post-hoc* analyses. The correlation between neural and non-neural components and clinical scales was calculated using Spearman's rank correlation coefficient (*r*_s_). *P* < 0.05 were considered significant. Correlation coefficients < 0.25 were considered as little to no, 0.25–0.50 as fair, 0.50–0.75 as moderate to good, and > 0.75 as good to excellent association (25).

## Results

### Population Characteristics

A total of 46 patients with chronic stroke and 30 healthy age-matched participants were included. One of the patients was not able to perform the NeuroFlexor measurements due to pain during wrist extension. Furthermore, data of three patients were excluded from the reliability analysis as their second measurement was missing due to technical problems. The main population characteristics are shown in Table 1.

**Table 1:** Demographic and clinical characteristics of the study population.

	**Stroke patients**	**Healthy subjects**
Participants (*n*)	46	30
Age, years (mean ± SD)	59.9 ± 10.0	59.0 ± 11.5
Gender, male/female (*n*)	31/15	14/16
Stroke type, iCVA/hCVA (*n*)	39/7	
Time post stroke, months (mean ± SD)	61.5 ± 76.5	
NIHSS score (mean ± SD)	4.8 ± 3.2	
Affected side, left/right (*n*)	26/20	
Dominant hand, left/right/ambidextrous (*n*)	3/42/1	0/30/0
MAS wrist flexor muscles (median [IQR])	1 [0–1.5]	
MAS finger flexor muscles (median [IQR])	1.5 (1–2)	
pROM affected/dominant wrist, ° (mean ± SD)	172.3 ± 20.1	167.9 ± 17.6
FM-UE (mean ± SD)	32.5 ± 18.7	
ARAT (mean ± SD)	21.0 ± 20.8	

*iCVA, ischemic stroke; hCVA, haemorrhagic stroke; NIHSS, National Institutes of Health Stroke Scale [range: 0–42]; MAS, modified Ashworth scale [range 0–4], (score 1+ is reported as 1.5); IQR, interquartile range; pROM, passive range of motion; FM-UE, Fugl-Meyer motor assessment of the upper extremity [range: 0–66]; ARAT, action research arm test [range: 0–57]*.

The majority of data was non-normally distributed, except for the VC in patients, the neural and non-neural components in healthy subjects, and the pROM of the wrist in both groups. The differences between NeuroFlexor measurements 1 and 2 were normally distributed for all three components.

### Test-Retest Reliability

An overview of the reliability parameters can be found in Table 2. The test-retest reliability (ICC) in the group of patients was excellent for the NC and EC (respectively, 0.93 and 0.95), and good for the VC (0.84). The SDC for the NC was 10.31 N, that for the EC 3.14 N, and that for the VC 0.53 N. Scatterplots of patients' test-retest data are presented in Supplementary File 2, and show a linear relationship between measurements 1 and 2 for all components. The plot for the EC shows three outliers with higher values (>mean value + 2 SD). Excluding these patients from analysis decreased the ICC to 0.75 (95% CI, 0.58–0.86), and changed the SDC from 3.14 to 3.02 N. Supplementary File 3 presents Bland-Altman plots, showing a distribution scattered around the mean difference of 0 for all components, which means there was no systematic difference between measurements 1 and 2.

**Table 2:** Reliability parameters of neural and non-neural components in patients with chronic stroke (*n* = 42).

	**ICC (95% CI)**	**Mean diff (SD)**	**SEM**	**SDC**
NC	0.93 (0.88–0.96)	0.27 (5.31)	3.72	10.31
EC	0.95 (0.92–0.98)	0.27 (1.60)	1.13	3.14
VC	0.84 (0.73–0.91)	−0.06 (0.27)	0.19	0.53

*ICC, intraclass correlation coefficient, two-way random-effects model for absolute agreement; 95% CI, 95% confidence interval; Mean diff, mean difference between measurements 1 and 2 (N); SEM, standard error of measurement (N); SDC, smallest detectable change (N); NC, neural component; EC, elastic component; VC, viscous component*.

### Construct Validity

The NeuroFlexor values of measurement 1 were used for the validation part of this study. Table 3 shows an overview of the median component values in healthy subjects and patients, stratified by MAS. Mann–Whitney *U*-tests revealed significantly higher NC, EC, and total resistance for patients compared to healthy subjects (*p* < 0.001) with significant differences between patients stratified by MAS score, and healthy subjects (NC, *p* < 0.001; EC, *p* < 0.001; VC, *p* < 0.05; total resistance, *p* < 0.001). *Post-hoc* analyses revealed significantly higher NC and EC values, and a lower VC value for patients with MAS = 0 compared to healthy subjects for all components (*p* < 0.03). Overall, the NC, EC, and VC gradually increased with MAS category, except for the VC for MAS categories 2 and 3.

**Table 3:** Neural and non-neural components of wrist hyper-resistance in healthy subjects and patients with chronic stroke.

			**Stroke, stratified by MAS**				
	**Healthy** ***n* = 30**	**Stroke** ***n* = 45**	**MAS 0** ***n* = 9**	**MAS 1** ***n* = 12**	**MAS 1+** ***n* = 11**	**MAS 2** ***n* = 7**	**MAS 3** ***n* = 6**
NC (N)	0.36 [−0.07 to 1.66]	11.54^*^ [4.84 to 20.99]	2.35^‡^ [0.86 to 10.88]	6.49 [3.58 to 15.75]	11.54 [8.96 to 20.29]	12.09 [8.93 to 26.64]	35.77 [21.97 to 47.21]
EC (N)	1.92 [1.41 to 2.72]	4.51^*^ [3.31 to 7.87]	3.15^†^ [2.32 to 3.99]	4.37 [2.88 to 6.99]	5.92 [4.43 to 8.12]	6.15 [4.38 to 8.10]	8.38 [3.92 to 22.11]
VC (N)	0.36 [0.15 to 0.63]	0.38 [0.04 to 0.67]	0.12^‡^ [−0.20 to 0.34]	0.22 [−0.20 to 0.57]	0.64 [0.38 to 1.04]	0.57 [0.11 to 0.75]	0.52 [−0.12 to 1.18]
Total (N)	2.61 [2.28 to 3.56]	17.43^*^ [8.53 to 28.96]	4.62^†^ [4.27 to 14.47]	12.71 [6.61 to 21.32]	18.74 [12.84 to 29.48]	18.64 [14.98 to 31.82]	43.28 [28.39 to 69.96]

Values are median [interquartile range]. MAS, modified Ashworth scale; NC, neural component; EC, elastic component; VC, viscous component; total, sum of three components. Level of significance of difference compared to healthy subjects (Mann-Whitney U test)

* p < 0.001;

† p < 0.01;

‡ *p < 0.05*.

A moderate to good significant positive correlation (*r*_s_ > 0.50, *p* > 0.01) was found between the NC, EC, and the total of components, and the MAS of both the wrist and finger flexor muscles (Table 4; Supplementary File 4). The NC and the total of components revealed a fair significant positive correlation with the Tardieu scale (*r*_s_ ≥ 0.30, *p* < 0.05). The NC, EC, and total of components showed fair significant negative correlation coefficients with the FM-UE and ARAT (*r*_s_ ≤ −0.38, *p* < 0.05).

**Table 4:** Spearman's rank correlation coefficients (*r*_s_) between neural and non-neural components of wrist hyper-resistance and clinical scales.

	**NC**	**EC**	**VC**	**Total**
Wrist flexor muscles				
- MAS	0.56^**^	0.49^**^	0.42^**^	0.57^**^
- TS Q	0.34^*^	0.21	0.26	0.30^*^
- TS R2-R1	0.36^*^	0.20	0.20	0.33^*^
Finger flexor muscles				
- MAS	0.60^**^	0.52^**^	0.37^*^	0.62^**^
- TS Q	0.24	0.23	0.30	0.26
- TS R2-R1	0.36^*^	0.26	0.19	0.37^*^
pROM wrist	0.12	−0.11	0.03	0.06
FM-UE	−0.41^**^	−0.47^**^	−0.29	−0.47^**^
ARAT	−0.38^*^	−0.42^**^	−0.24	−0.44^**^

* p < 0.05;

** *p < 0.01; NC, neural component; EC, elastic component; VC, viscous component; total, sum of three components; MAS, modified Ashworth scale; TS Q, Tardieu scale, quality score; TS R1-R2, Tardieu scale, range R2-R1; pROM, passive range of motion; FM-UE, Fugl-Meyer assessment of the upper extremity; ARAT, action research arm test*.

## Discussion

We have investigated the test-retest reliability and construct validity of the easily applicable and commercially available NeuroFlexor for the quantification of neural and non-neural components of hyper-resistance in the wrist joint in a group of 46 patients with chronic stroke with initial upper limb impairments, using a test-retest design with a single experimental session. The reliability for the neural and elastic components was excellent, and good reliability was found for the viscous component. Despite the promising reliability results, the SDC for all components was large compared to the median values (70–140% of the median). The significantly greater NC and EC in patients compared to healthy subjects, as well as the positive association of NC and EC with the MAS scores of both the wrist and fingers flexor muscles, the positive association of NC with the Tardieu scale, and the negative association with the motor performance of the arm, suggest that the NeuroFlexor method has good construct validity.

### Reliability

In the previous study by Gäverth et al. (15), equivalent ICC values were found for test-retest reliability (NC, 0.93; EC, 0.84; VC, 0.89) in a comparable group of patients with chronic stroke. Comparison of measurement error is difficult, however, as Gäverth et al. (15) used a logarithmic transformation to cope with the heteroscedasticity of their data, whereas there was no need for log transformation in our data. The calculated SDC scores allow for an easier interpretation in clinical practice. Moreover, reliability may have been exaggerated in the study by Gäverth et al. (15), because a constant value was added to the raw data to compensate for negative values of the measured components to allow logarithmic transformation, which influences the variances (26).

The relatively high SDC values we found, with good to excellent ICC values, can be explained by the heterogeneity of the study population we included, as ICC is strongly influenced by the variability between patients, whereas this variability is not included in the calculation of the SDC. A real change that could be measured (SDC value) is only a little smaller than the median values for the NC and EC, and even higher than the median value for the VC. This suggests that the NeuroFlexor is a reliable method for research purposes at group level and to differentiate between patients, but is less capable of detecting changes within individual patients over time. When monitoring a treatment effect in a single patient, a decrease of at least the SDC has to be achieved by the intervention to be interpreted as a real treatment effect. To use the NeuroFlexor for individual treatment decisions and evaluation, the method needs further improvement in terms of standardization.

To our knowledge, the NeuroFlexor is the first instrument available for the quantification of the neural and non-neural components of hyper-resistance without the assessment of electromyography (EMG) of the muscles involved. This means that this device is more feasible for use in clinical practice. Other measurement setups which use EMG for the quantification of components of hyper-resistance, have shown comparable or even poorer reliability values in terms of ICC and SDC (13, 27–29). Adding EMG to the NeuroFlexor measurement would presumably not improve the reliability. However, previous research showed that torque-related biomechanical parameters alone are less valid to describe the construct of spasticity than EMG-related parameters (30, 31). Moreover, the quantification of the neural component will always remain challenging, as spasticity is known to be variable in time and dependent on multiple factors such as posture, temperature, and emotional status (32). To account for natural fluctuations and to decrease SDC, repeated measurements within one session might be a solution (33). However, this will also reduce the clinical applicability.

### Construct Validity

The NeuroFlexor is able to discriminate between healthy subjects and patients, even when classified as MAS = 0 for the wrist and finger flexor muscles. According to the pathology of stroke, patients show increased neural and non-neural hyper-resistance in the wrist (1, 2). The difference in the neural component we found between healthy subjects and patients in the MAS = 0 category emphasizes the presence of hyper-excitability of the stretch reflex in all patients, even without clinical hyper-resistance (34). Additionally, the variances in neural and non-neural components of hyper-resistance were larger in patients compared to healthy subjects, reflecting the heterogeneity and therefore emphasizing the importance of individualized assessments for treatment decisions.

As expected, the total resistance to passive movement, as measured with the NeuroFlexor, was higher for patients in higher MAS categories. Both the NC and EC showed a good association with the MAS score, which emphasizes the criticism that has been made about the MAS, that it is not able to differentiate between these components and is influenced by both. A fair positive association was found between the NC and TS, which is supposed to be a more valid measure of the velocity-dependent spasticity (9). The expected association between the EC and the pROM of the wrist was not found, probably because we used pROM restriction as an exclusion criterion for study participants. Performing the NeuroFlexor measurements requires a wrist extension with extended fingers of at least 40°. However, there was a significant difference in EC between healthy subjects and patients. If the NeuroFlexor is to be used in the future as a treatment evaluation method following, for example, botulinum toxin injections, the device and model need to be adapted for patients with pROM restrictions, as these are most often treated.

Our findings suggest that the NeuroFlexor shows concurrent validity against the MAS and the Tardieu scale. However, future research is necessary to further validate its ability to distinguish the neural and non-neural components of hyper-resistance. This could be achieved by comparing the NeuroFlexor method with other instrumented assessment techniques, and more fundamental studies are needed to validate the different components of hyper-resistance. Furthermore, the responsiveness of the NeuroFlexor measurements to different treatments should be evaluated in relation to the SDC and minimal important change. The neural component can be influenced by treatments such as botulinum toxin and baclofen, whereas the non-neural component could be influenced by casts, splints, or orthopedic surgery.

Interestingly, both the NC and EC have a fair negative association with the FM-UE and ARAT scores. Increased neural and non-neural hyper-resistance in the wrist is associated with poorer motor performance and arm-hand capacity. Further longitudinal studies are needed to investigate the development of hyper-resistance post stroke and its interaction with synergy-dependent motor recovery as measured with FM-UE, as well as the recovery of quality of movement. Knowledge of the time course of development of post-stroke hyper-resistance and its interaction with motor recovery is very important to better understand the neurophysiological changes that occur when patients recover.

### Study Limitations

This study did not address interrater reliability. However, adding another source of variance by a second observer would likely increase the measurement error, and would therefore not change our conclusion about the limited suitability of the NeuroFlexor for individual treatment evaluation. The ICC of the EC might have been inflated by three outliers observed in the scatterplot, which emphasizes the impact of heterogeneity of the study population on the ICC value. Due to a lack of an appropriate golden standard, it is not possible to study the criterion validity of the NeuroFlexor, and construct validity is to date the only possibility. Additionally, assessment of construct validity was difficult due to the fact that the constructs of the commonly used clinical scales MAS and TS are ambiguous. It is important to note that the correlation between the neural and non-neural components and the MAS confirms the inadequacy of this manual test, rather than highlighting the construct validity of the NeuroFlexor. Although we tried to limit influences on hyper-resistance, we were not able to estimate the variance in wrist hyper-resistance between the two measurement sessions due to lack of a golden standard. As the NeuroFlexor provokes a wrist movement with extended fingers in a small range around the neutral position, both the wrist flexor muscles as well as the finger flexor muscles lengthen and contribute to the neural and non-neural components. To address test-retest reliability, we strictly adhered to a fixed position of the hand and fingers in the device. Future studies, however, may use different finger positions with respect to wrist and finger flexor muscle lengths, depending on the research or clinical question and the mechanical constraints of the device. Furthermore, the wrist was extended at two arbitrarily selected velocities (5 and 236°/s, respectively), which are assumed to be well below and well above expected reflex threshold velocity (35). It should be acknowledged that the current linear approach does not address non-linear features as length and velocity dependent threshold of the stretch reflex (36). Lastly, due to restrictions in the measurement setup of the NeuroFlexor, we had to exclude patients with passive wrist extension limited to <40°, which will have affected the association between the EC and the pROM, and limits the generalization of our results.

## Conclusions

The NeuroFlexor is a reliable instrument for the quantification of neural and non-neural components of hyper-resistance in the wrist joint at group level in patients with chronic stroke who exhibit initial upper limb impairments. The instrument showed good to excellent reliability for both neural and non-neural components. However, high SDC values make it difficult to use this technique for individual treatment decisions. The NeuroFlexor method appears to be construct valid against clinical scales, although the validity of the different components needs further investigation.

Overall, the NeuroFlexor can replace current clinical scales to evaluate wrist hyper-resistance for research purposes at group level and to differentiate between patients. For individual use in clinical practice, however, the NeuroFlexor needs further improvement in terms of measurement error and applicability in patients with decreased wrist range of motion.

## Data Availability

The dataset analyzed during the current study is available from the corresponding author on reasonable request.

## Ethics Statement

The study has been approved by the Medical Ethical Committee of the VU University Medical Center, Amsterdam, Netherlands (protocol number 2014.140). In accordance with the Declaration of Helsinki (2013), all participants gave written informed consent.

## Author Contributions

AA contributed to the design of the study, recruited the patients, conducted the experiments, performed data analysis, and drafted the manuscript. EvW, IvdP, GK, and CM contributed to the design of the study, took part in the interpretation of the data, and revised the manuscript. All authors contributed to the final version of the report.

### Conflict of Interest Statement

The authors declare that the research was conducted in the absence of any commercial or financial relationships that could be construed as a potential conflict of interest.

## Abbreviations

ARAT: action research arm test
EC: elastic component
FM-UE: Fugl-Meyer motor assessment of the upper extremity
MAS: modified Ashworth scale
MMSE: mini mental state examination
NC: neural component
NIHSS: National Institutes of Health Stroke Scale
pROM: passive range of motion
SDC: smallest detectable change
TS: Tardieu scale
VC: viscous component.
