# Supplementary material for: Measurement Properties of the NeuroFlexor Device for Quantifying Neural and Non-neural Components of Wrist Hyper-Resistance in Chronic Stroke
Source: Front Neurol. 2019 Jul 3;10:730. doi: 10.3389/fneur.2019.00730 (PMC6618514; doi:10.3389/fneur.2019.00730)
Supplement: Supplementary file 1 [file Data_Sheet_1.docx]

Supplementary Material

# Biomechanical model NeuroFlexor

In the biomechanical model of the NeuroFlexor, previously described by Lindberg et al. (1), the total measured resisting force (F*_m_*) during passive wrist extension is a summation of passive elastic force (F*_p_*), viscous force (F*_v_*), reflexive force (F*_r_*), and inertial forces of the limb and the moving parts of the device (F*_in_*), described as:

$F_{m}(\theta) = F_{p}(\theta) + F_{v}(\theta) + F_{r}(\theta) + F_{in}(\theta)$, where θ denotes a specific angle.

In the model, three force points in the resistance trace of the slow and fast displacements are used to estimate the different components of the total measured passive force. Two force points are defined within the fast passive wrist extension movement (236°/s): P1, the initial peak in resistance, and P2, the late peak in resistance (Supplementary Figure 1A). One force point (P3) is defined at the end position of the slow wrist extension movement (5 °/s) (Supplementary Figure 1B).

The **inertia component (IC)** corresponds to the force resisting the acceleration of the hand and is calculated in the model as:

$IC = m \times a$,

where *m* is the mass of the hand and the movable platform, and *a* is the angular acceleration (21 m/s^2^). The mass of the hand is estimated to be 0.6% of the total body weight.

The **elastic component (EC)** is a length-dependent resisting force which increases when the muscles are stretched, with an exponential increase when the muscle is stretched close to its end range. The EC is recorded 1 second after the end of the slow movement. The EC corresponds to P3, i.e. the fully stretched position during the slow movement (Supplementary Figure 1B).

The **viscous component (VC)** is velocity-dependent. Lindberg et al. (1) assumed that the viscous resistance is highest during the initial acceleration and continues at a lower level during further extension movement. To calculate the viscous component, first, the early viscous component (VC_P1_) is calculated.

${VC}_{P1}={Total force}_{P1} - IC$,

where Total force_P1_ is the measured force at P1 (Supplementary Figure 1A), and IC the inertial component calculated as above. Since there is a comparatively stable relationship between the early and late viscosity, Lindberg et al. assumed that the late viscosity is approximately 20% of the early viscosity.

$VC = {VC}_{P1}\times0.2$.

Finally, P2 is defined as the late force peak during the fast wrist extension movement (Supplementary Figure 1A) and consists of the neural, viscous and elastic component together. The **neural component (NC)** is estimated by:

$NC = {Total force}_{P2}- (EC + VC)$.


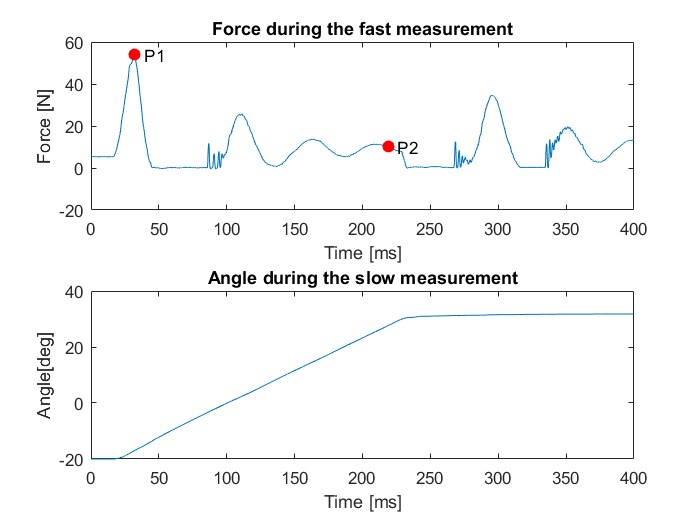


**Supplementary Figure 1A.** Fast movement NeuroFlexor


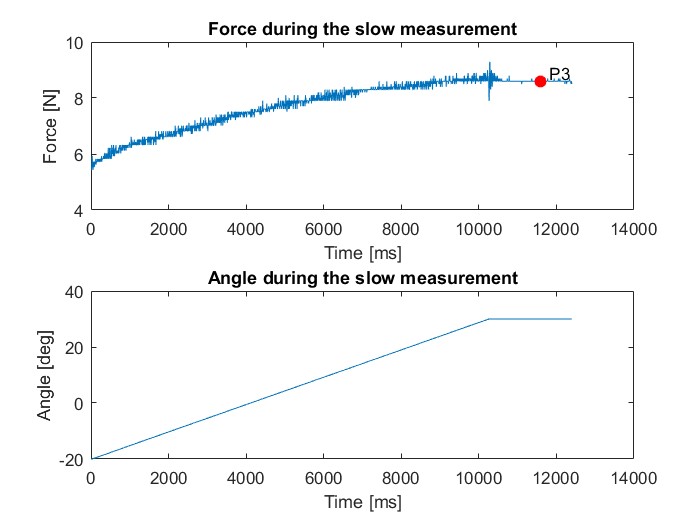


**Supplementary Figure 1B.** Slow movement NeuroFlexor

**References**

Lindberg PG, Gaverth J, Islam M, Fagergren A, Borg J, Forssberg H. Validation of a new biomechanical model to measure muscle tone in spastic muscles. *Neurorehabil Neural Repair* (2011) 25:617-25.

# Scatterplots

A.
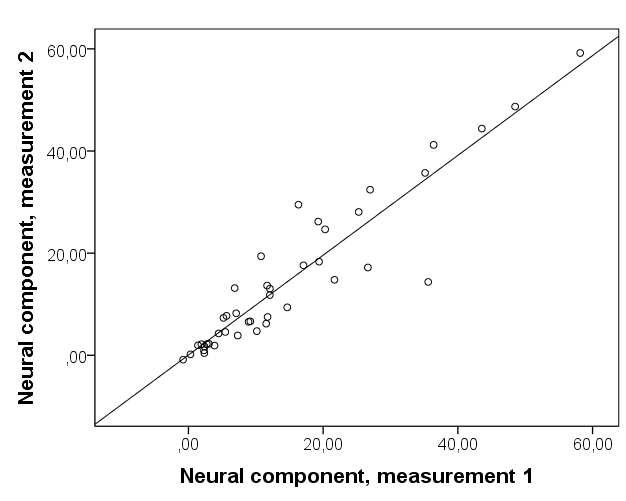


B.
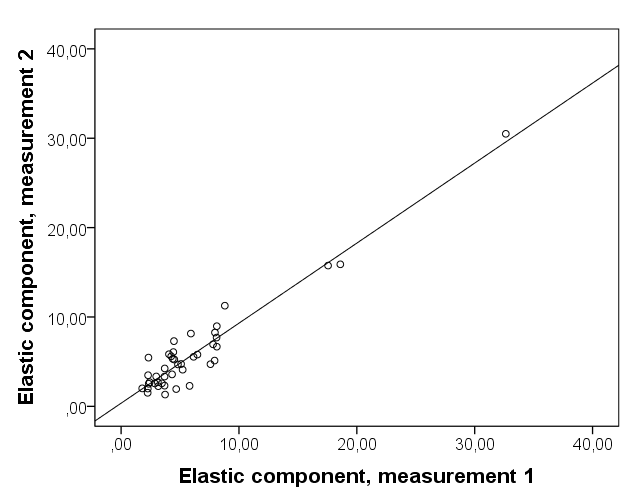


C.
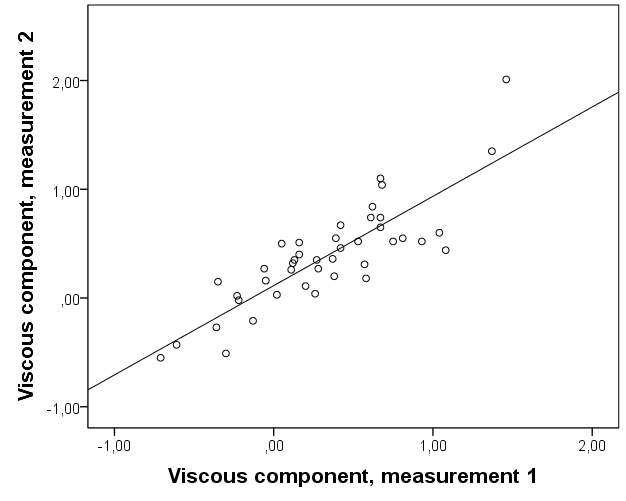


**Supplementary Figure 2.** Scatterplots, measurement 1 against measurement 2 in patients with chronic stroke. (A) Neural component. (B) Elastic component. (C) Viscous component.

# Bland-Altman plots

A.
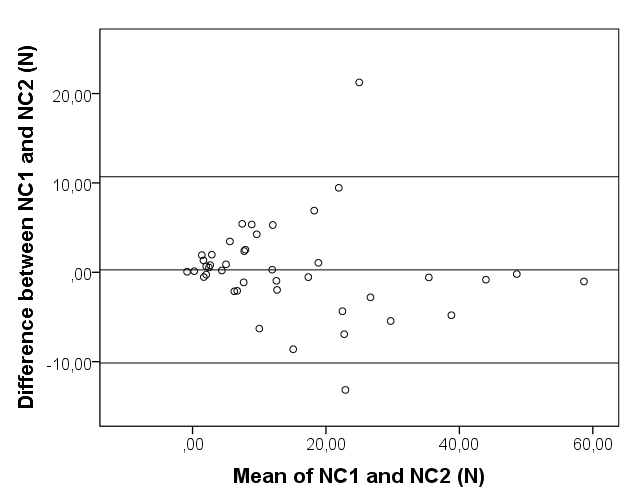


B.
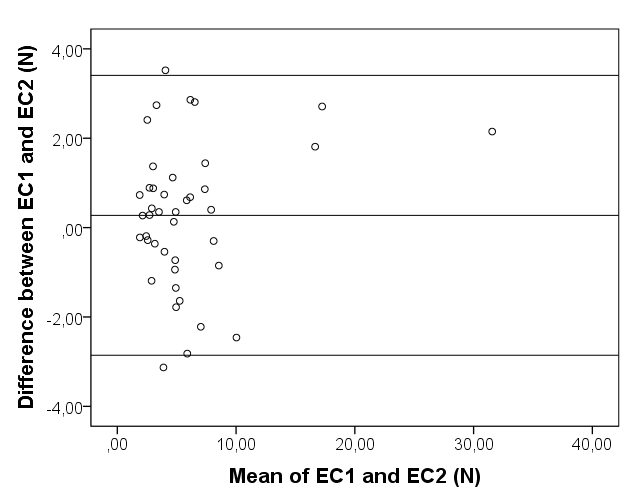


C.
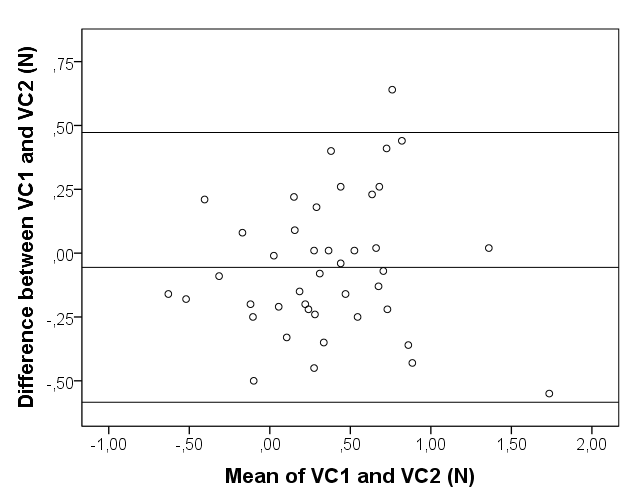


**Supplementary Figure 3.** Bland-Altman plots for neural, elastic, and viscous component in patients with chronic stroke. (A) Neural component. (B) Elastic component. (C) Viscous component.

# Scatterplots of correlation between neural and non-neural components of wrist hyper-resistance and clinical assessments

#
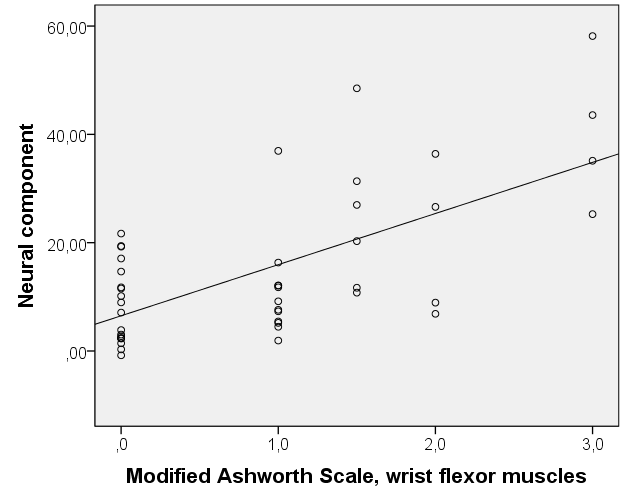

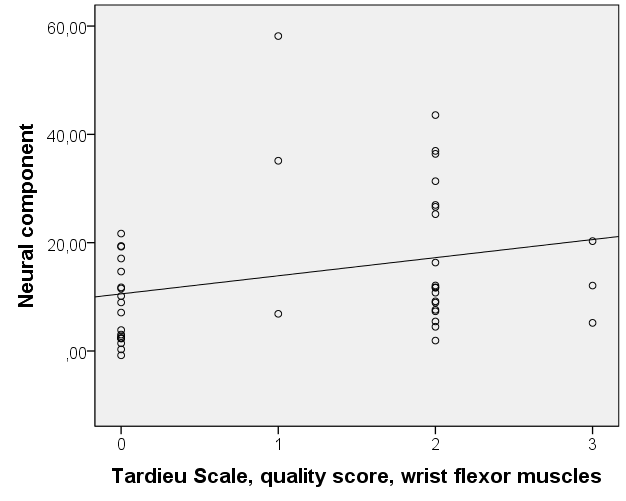

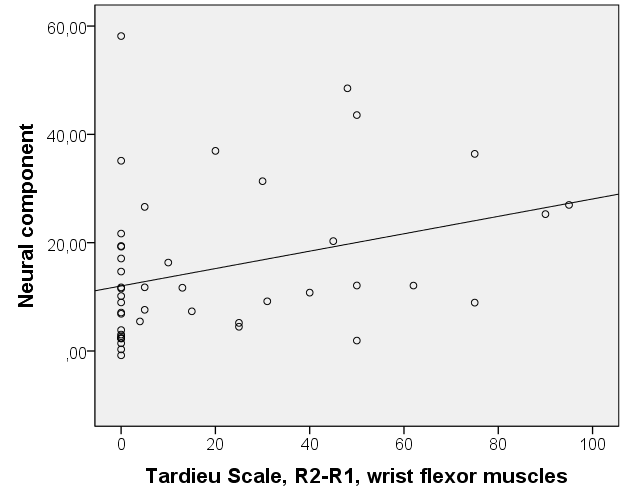


#
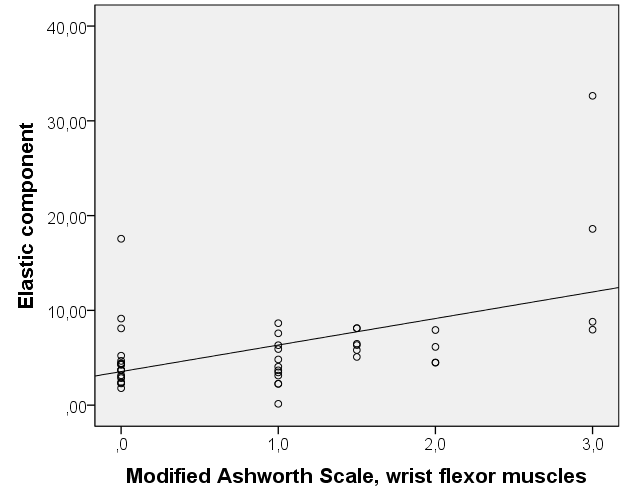

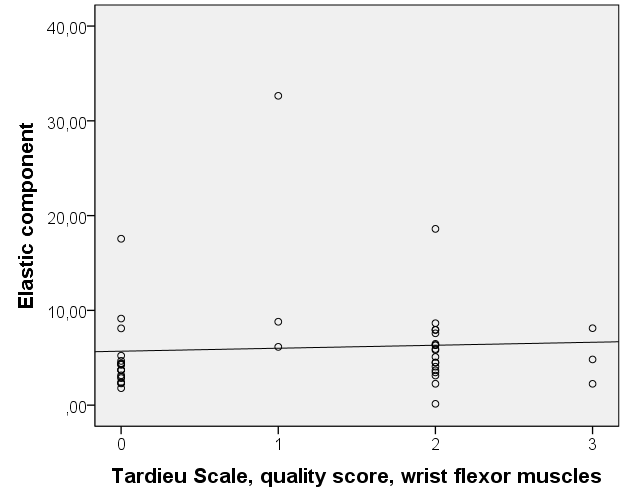

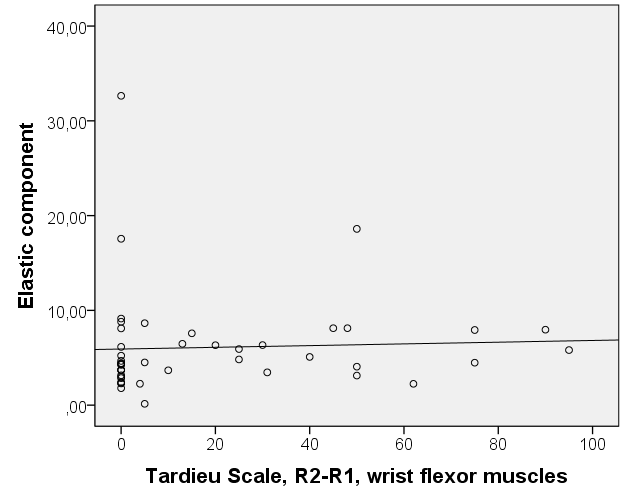


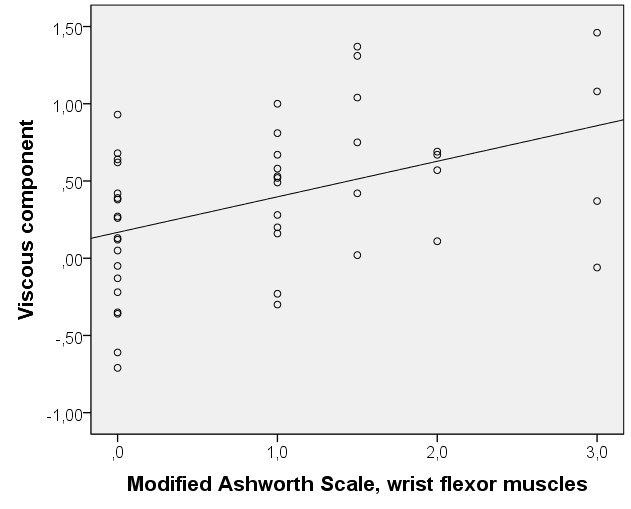

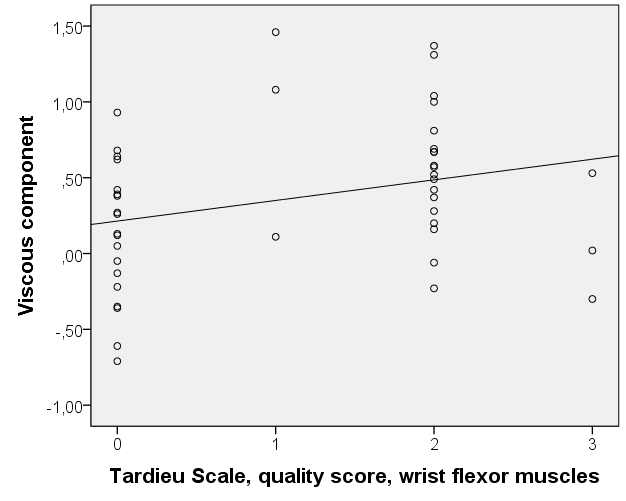

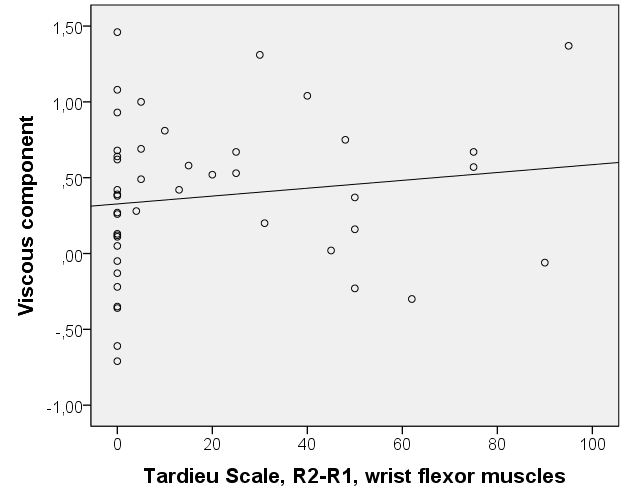


#
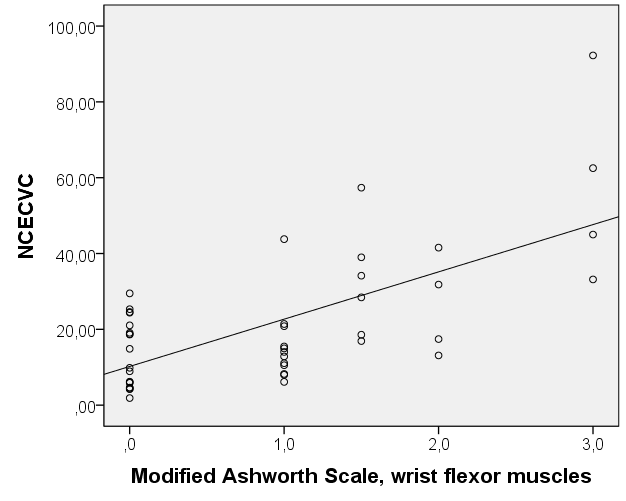

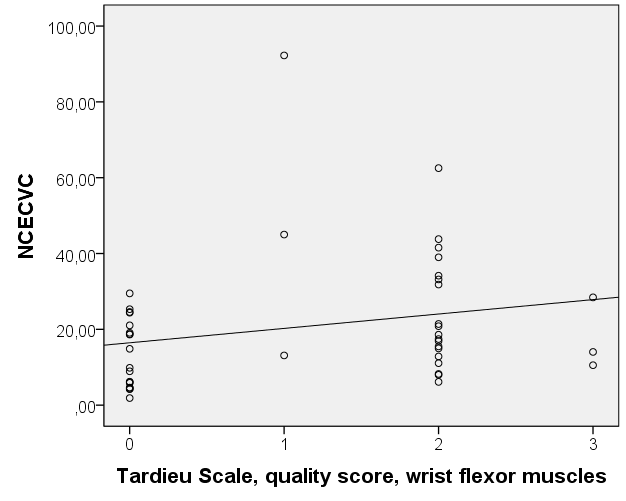

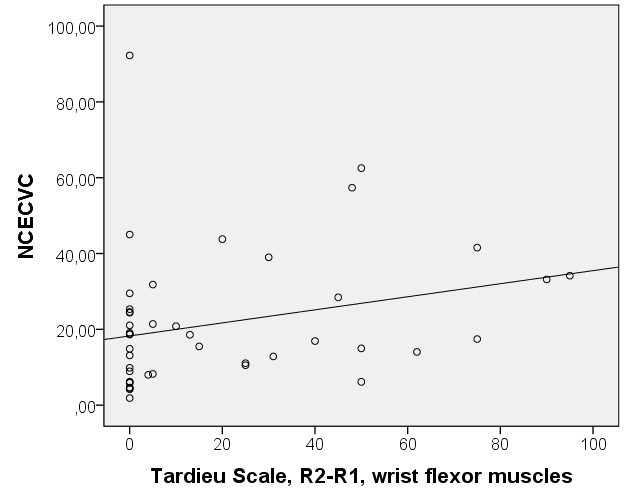


# Supplementary Figure 4A. Correlation between modified Ashworth scale, Tardieu scale quality score, and Tardieu scale R2-R1 of wrist flexor muscles, and neural and non-neural components.


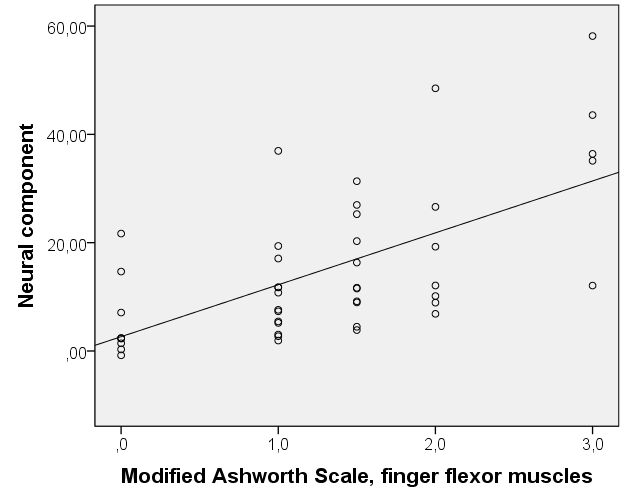

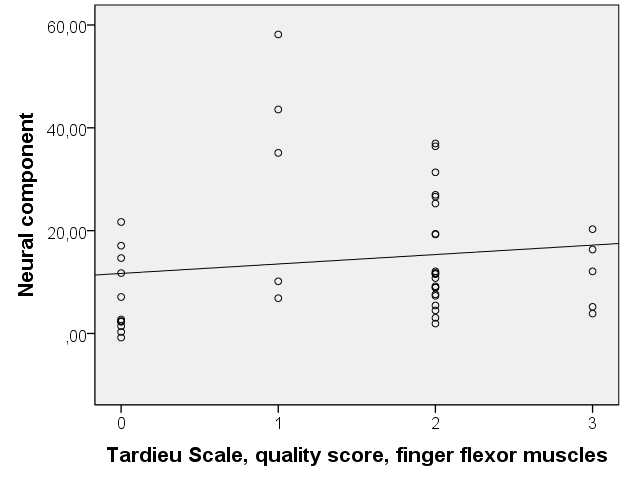

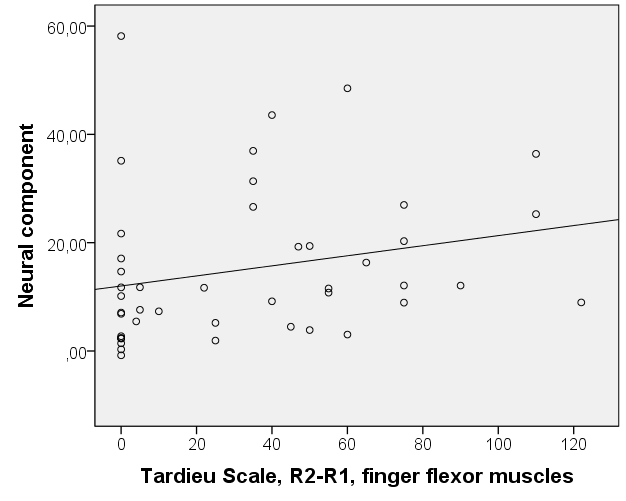

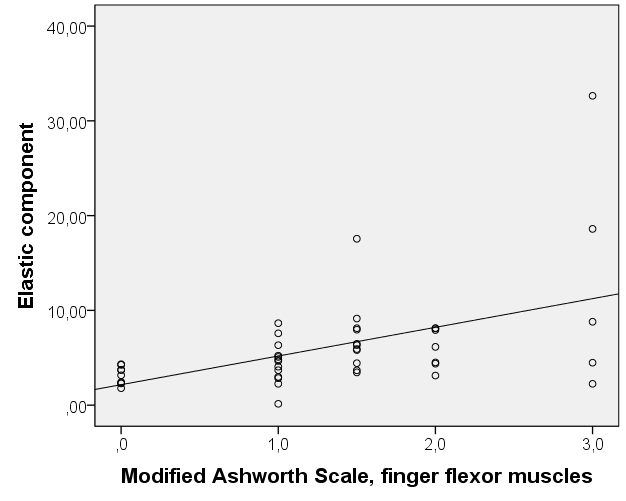

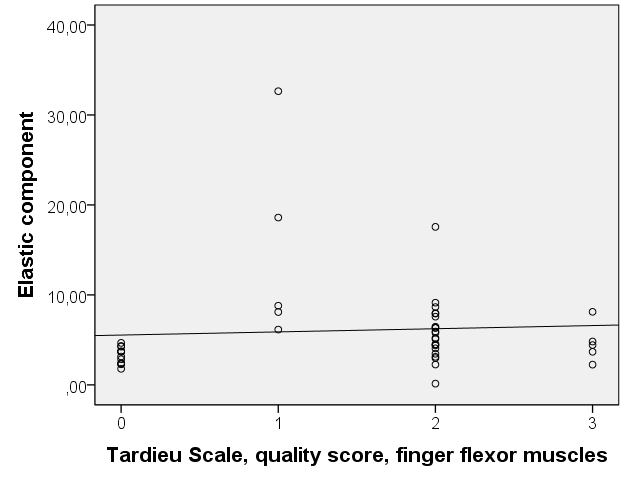

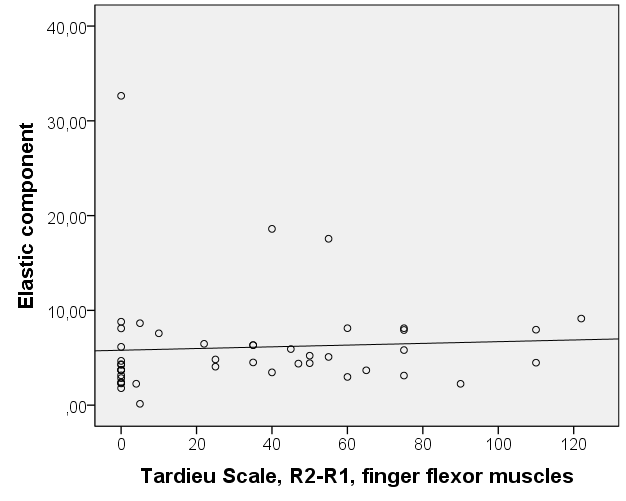


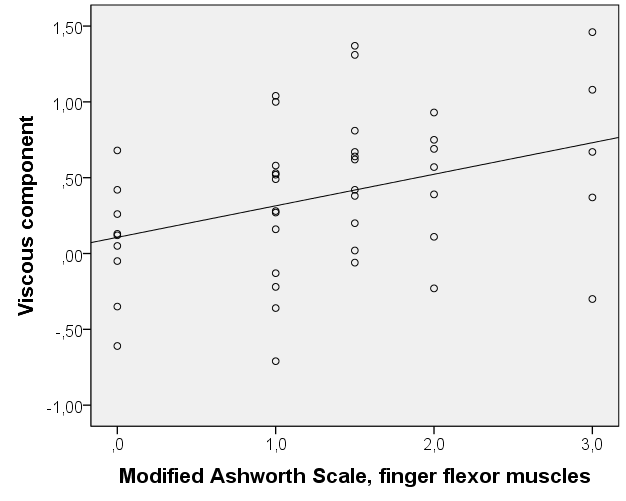

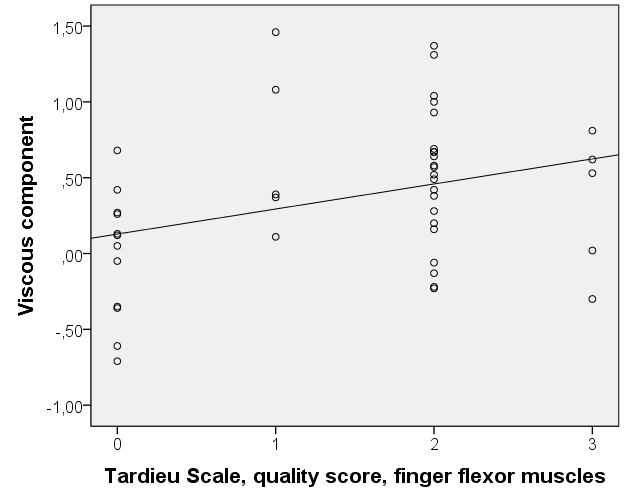

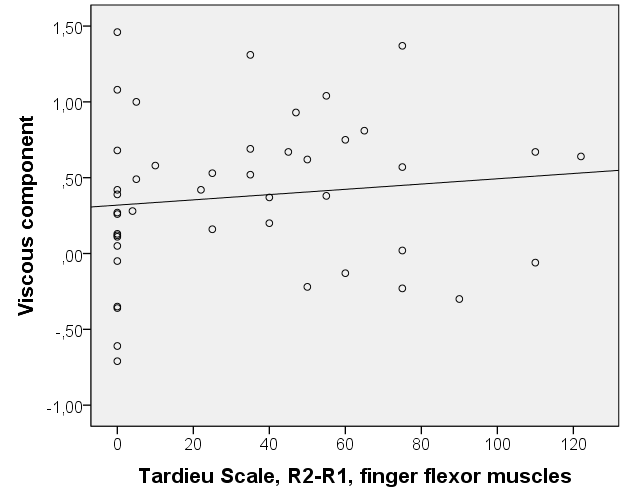


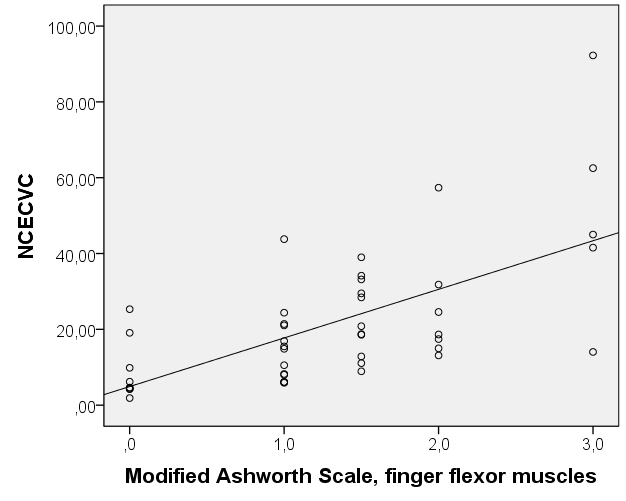

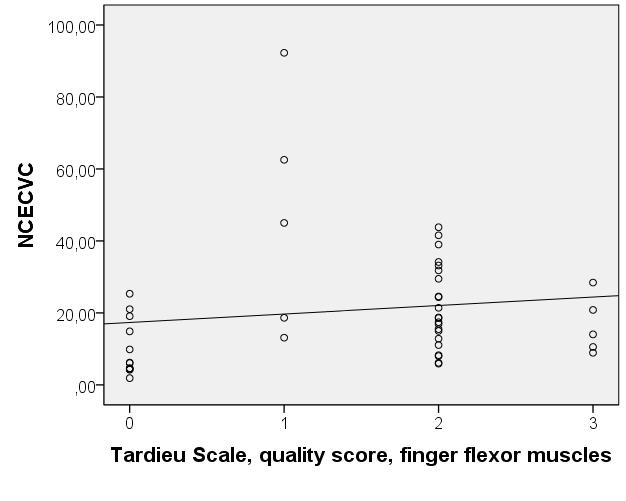

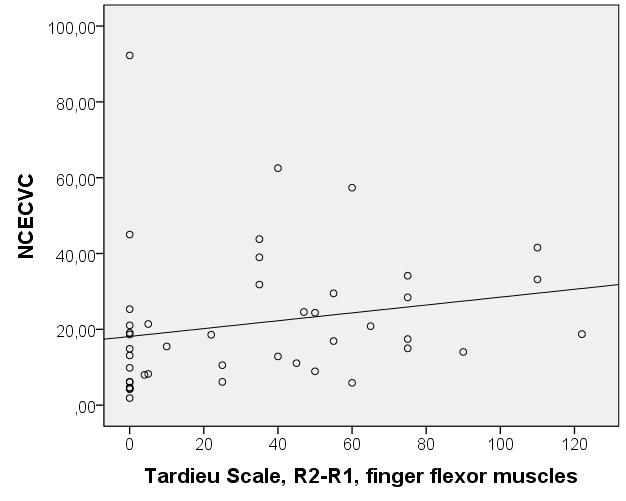


**Supplementary Figure 4B.** Correlation between modified Ashworth scale, Tardieu Scale quality score, and Tardieu Scale R2-R1 of finger flexor muscles, and neural and non-neural components.


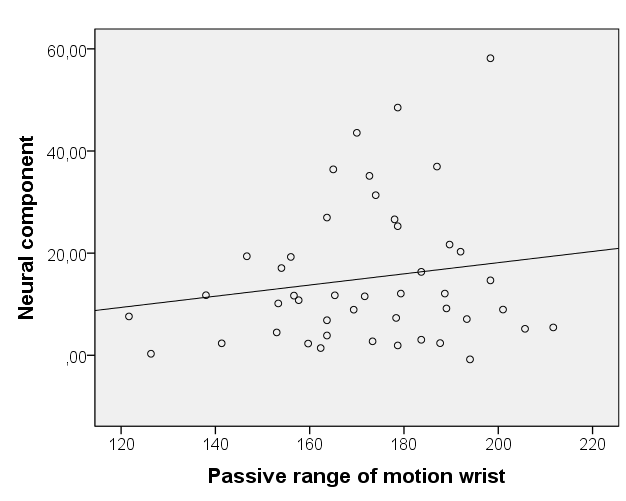

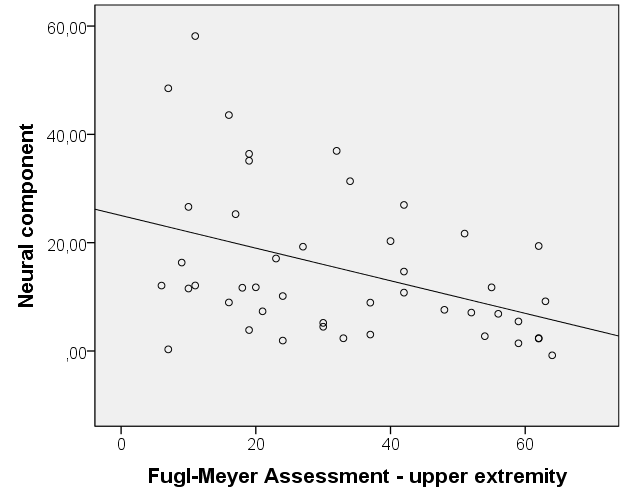

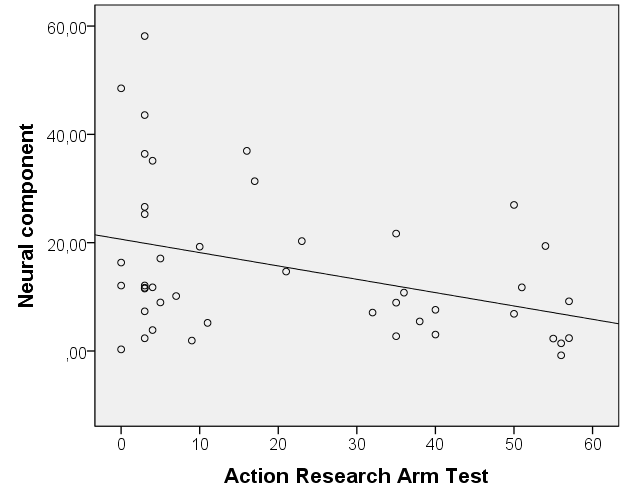

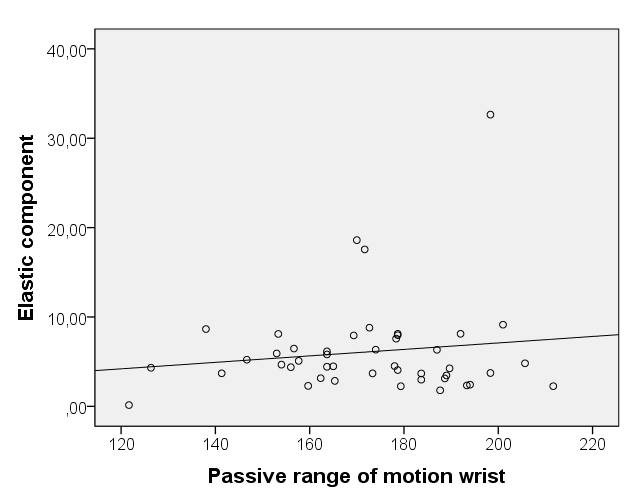

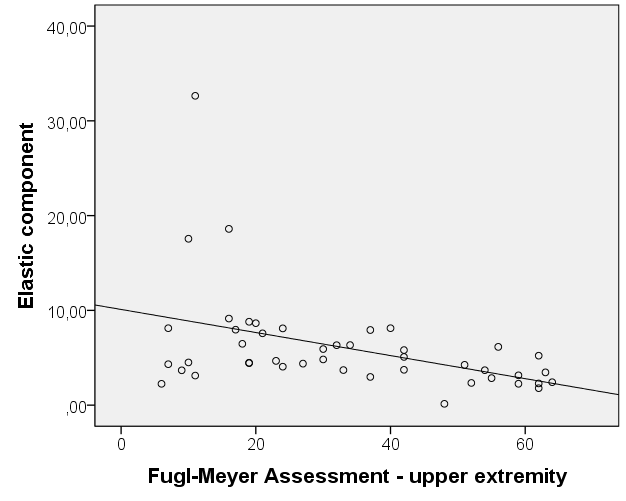

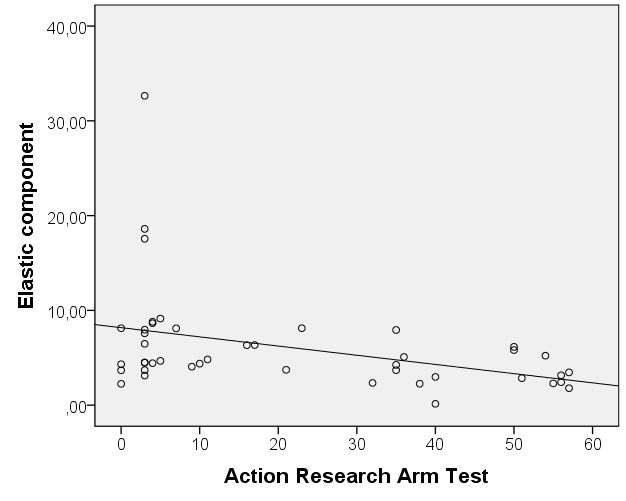


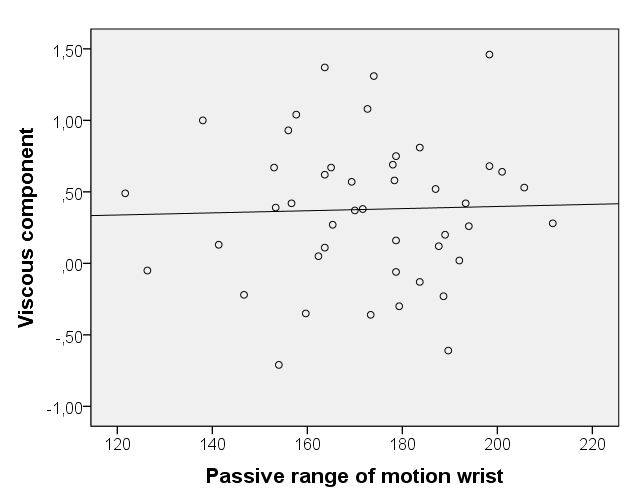

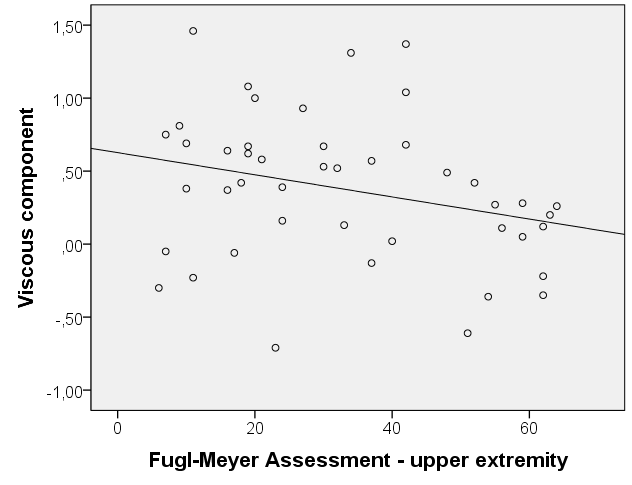

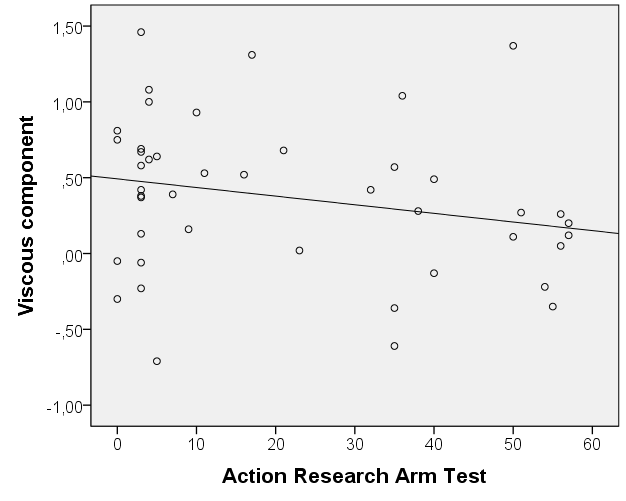


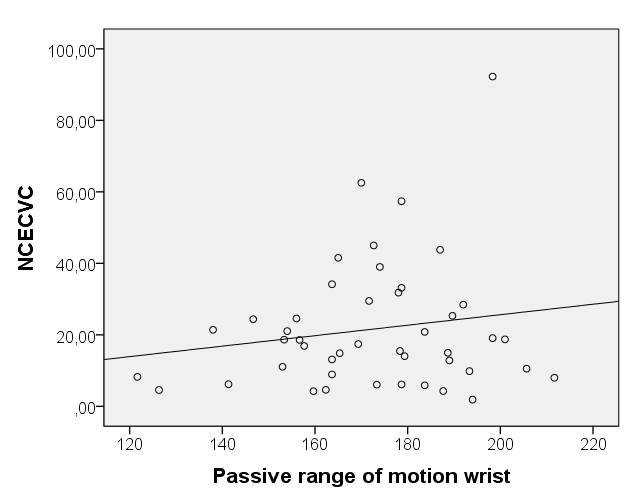

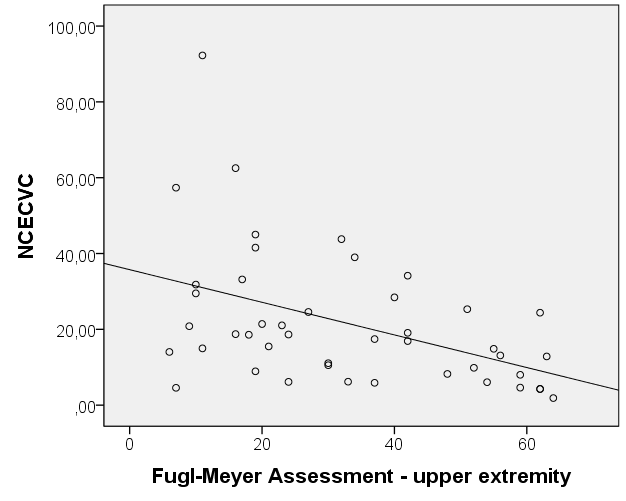

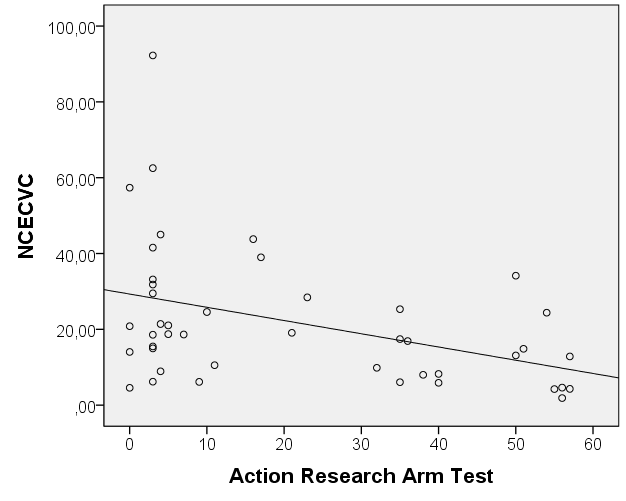


**Supplementary Figure 4C.** Correlation between passive range of motion of the wrist, Fugl-Meyer assessment of the upper extremity, and action research arm test, and neural and non-neural components.
